# Supplementary material for: SRSF5 Regulates the Expression of BQ323636.1 to Modulate Tamoxifen Resistance in ER-Positive Breast Cancer
Source: Cancers (Basel). 2023 Apr 13;15(8):2271. doi: 10.3390/cancers15082271 (PMC10136643; doi:10.3390/cancers15082271)
Supplement: Supplementary file 1 [file cancers-15-02271-s001.zip › cancers-2221229-supplementary.pdf]

Figure S1

DNA sequence of exon 10 to exon 12 of NCOR2

GAGCAGAAGTTCTGCCAGCGCTATGACCAGCTCATGGAGGCCTGGGAGAAGAAGGTGGAGCGCATCGAGAACAACC
CCCGGCGGCGGGCCAAGGAGAGCAAGGTGCGCGAGTACTACGAGAAGCAGTTCCCTGAGATCCGCAAGCAGCGCGA
GCTGCAGGAGCGCATGCAGAGGGTGGGCCAGCGGGGCAGTGGGCTGTCCATGTCGGCCGCCCGCAGCGAGCACGA
GGTGTCAGAGATCATCGATGGCCTCTCAGAGCAGGAGAACCTGGAGAAGCAGATGCGCCAGCTGGCCGTGATCCCGC
CCATGCTGTACGACGCTGACCAGCAGCGCATCAAGTTCATCAACATGAACGGGCTTATGGCCGACCCCATGAAGGTGTA
CAAAGACCGCCAGGTCATGAACATGTGGAGTGAGCAGGA**GAAGGAGACCTTCCGGGAGAA**

Red: exon 11 (91 bp)

Blue: target sites of NCOR2-BQ splicing forward and reverse primers

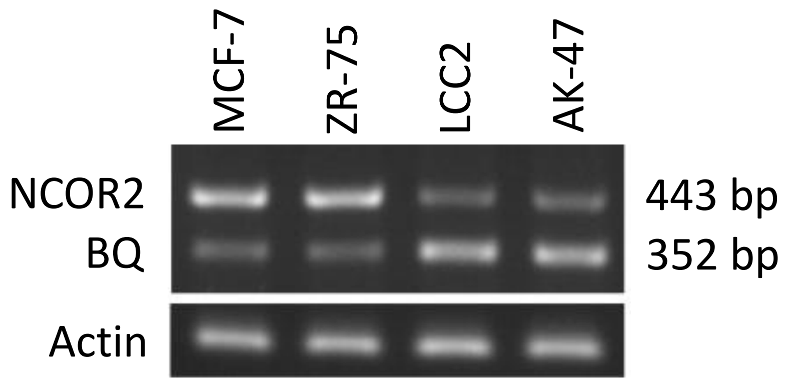

Figure S2

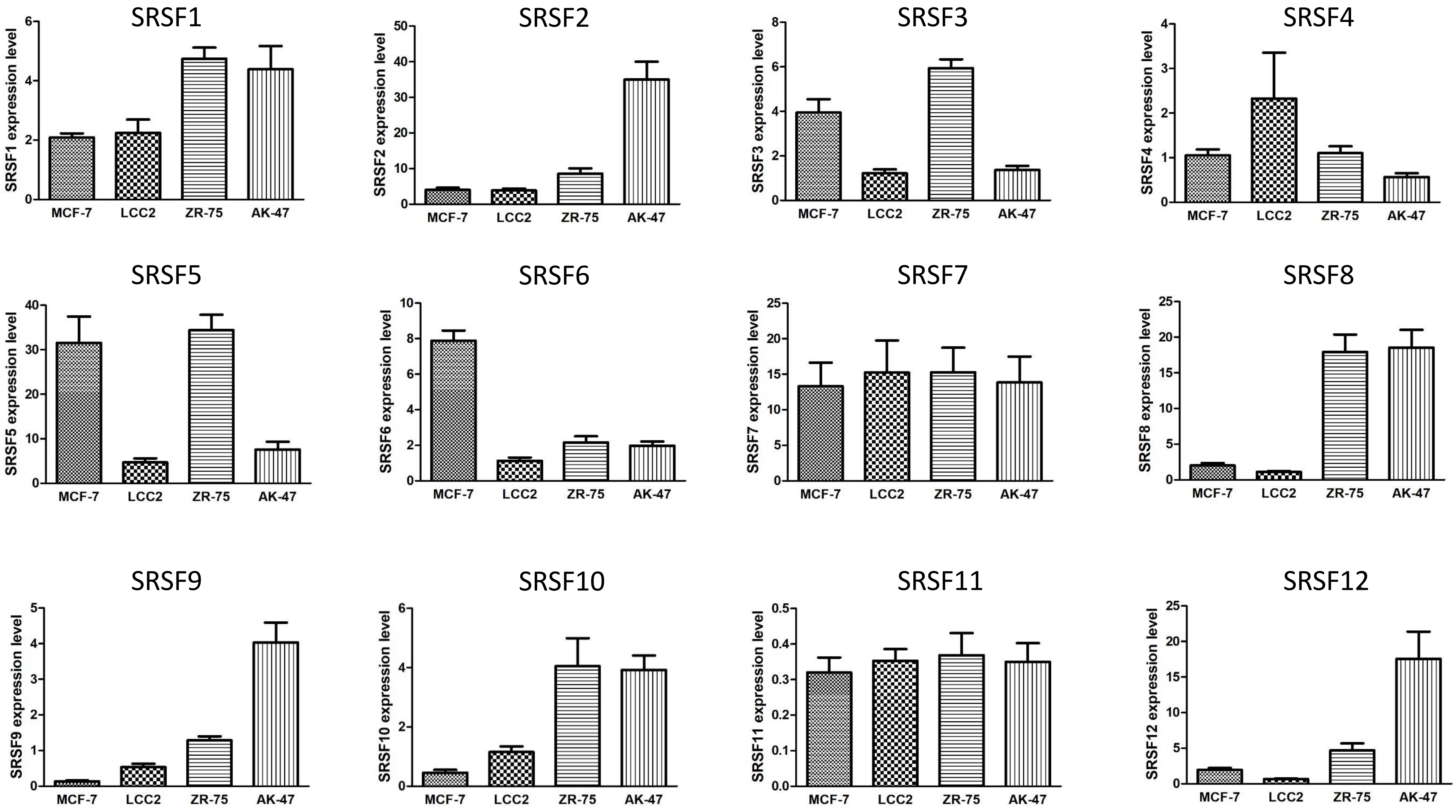
SRSF1
SRSF1 expression level
SRSF2
SRSF2 expression level
SRSF3
SRSF3 expression level
SRSF4
SRSF4 expression level
SRSF5
SRSF5 expression level
SRSF6
SRSF6 expression level
SRSF7
SRSF7 expression level
SRSF8
SRSF8 expression level
SRSF9
SRSF9 expression level
SRSF10
SRSF10 expression level
SRSF11
SRSF11 expression level
SRSF12
SRSF12 expression level
MCF-7
LCC2
ZR-75
AK-47

Figure S3

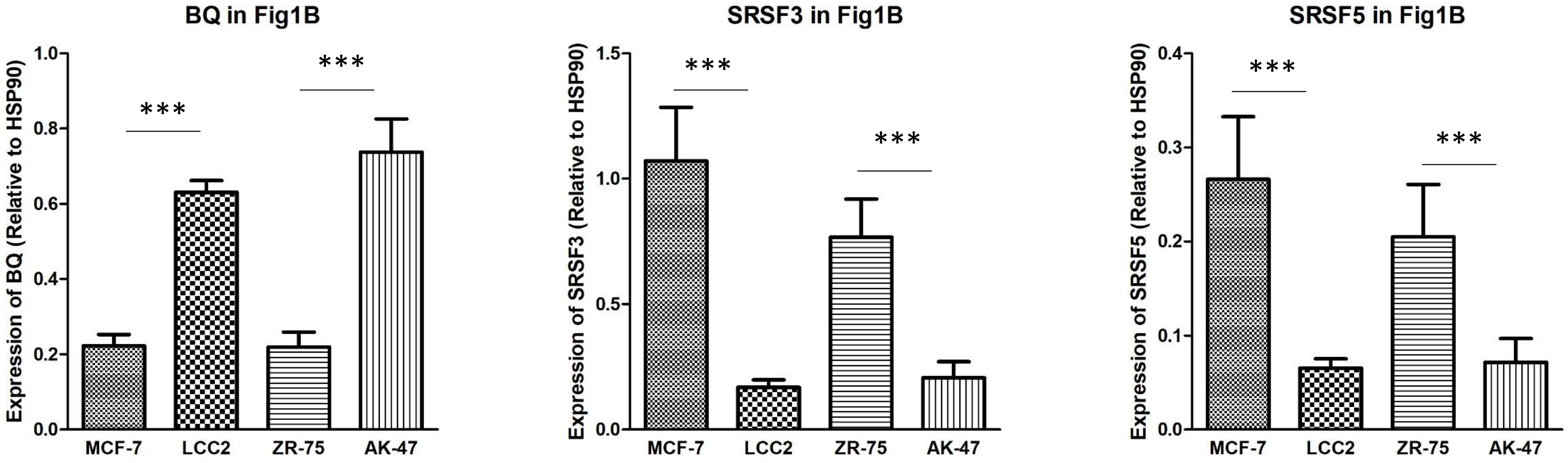
BQ in Fig1B
Expression of BQ (Relative to HSP90)
1.0
0.8
0.6
0.4
0.2
0.0
***
***
MCF-7
LCC2
ZR-75
AK-47
SRSF3 in Fig1B
Expression of SRSF3 (Relative to HSP90)
1.5
1.0
0.5
0.0
***
***
MCF-7
LCC2
ZR-75
AK-47
SRSF5 in Fig1B
Expression of SRSF5 (Relative to HSP90)
0.4
0.3
0.2
0.1
0.0
***
***
MCF-7
LCC2
ZR-75
AK-47

Figure S4

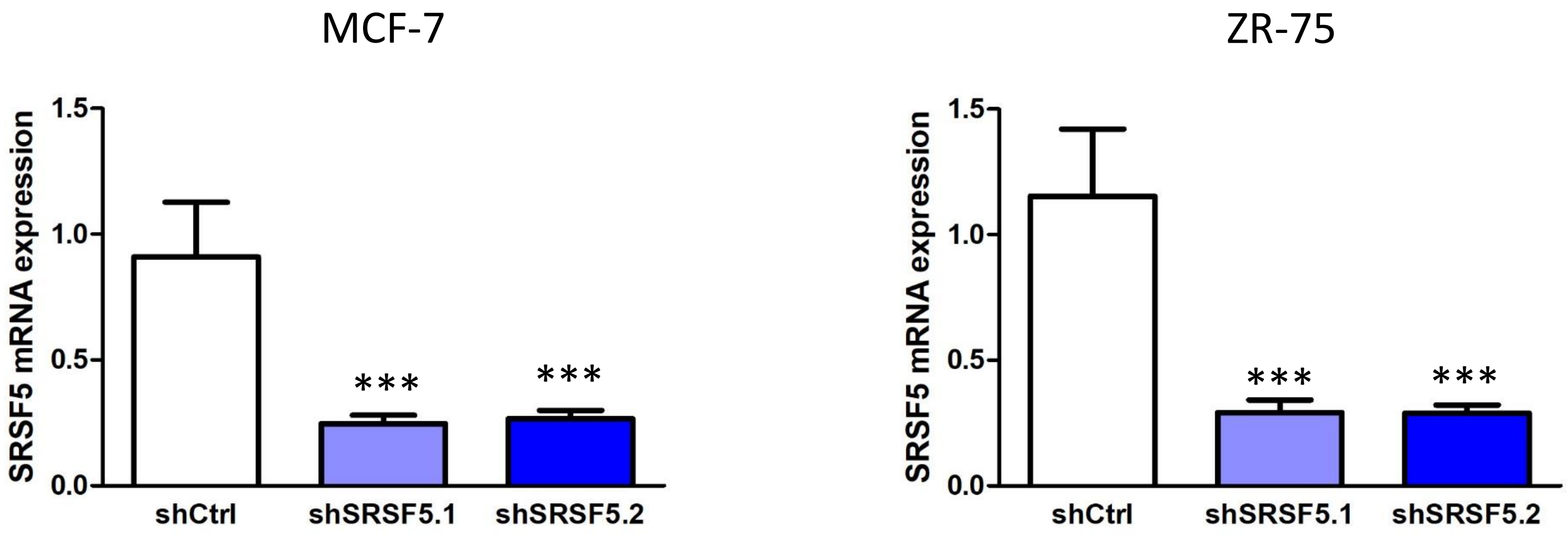
MCF-7
SRSF5 mRNA expression
1.5
1.0
0.5
0.0
***
***
shCtrl
shSRSF5.1
shSRSF5.2
ZR-75
SRSF5 mRNA expression
1.5
1.0
0.5
0.0
***
***
shCtrl
shSRSF5.1
shSRSF5.2

Figure S5

BQ/GADPH MCF-7 Fig 1C

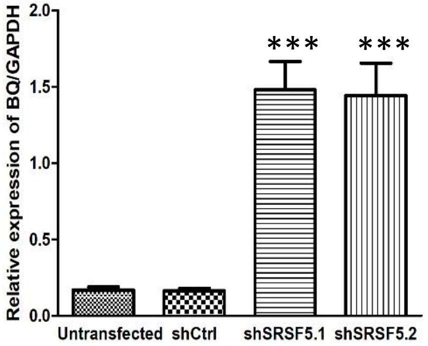

SRSF5/GADPH MCF-7 Fig 1C

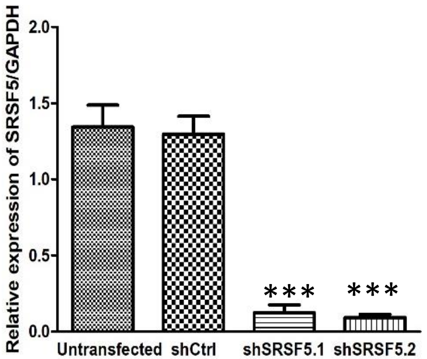

BQ/GADPH ZR-75 Fig 1C

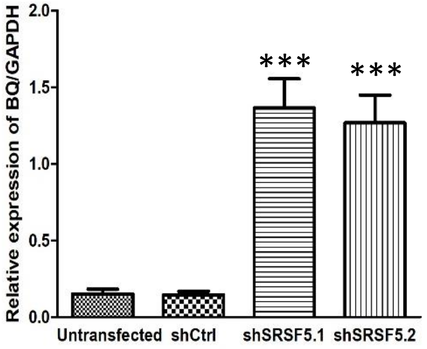

SRSF5/GADPH ZR-75 Fig 1C

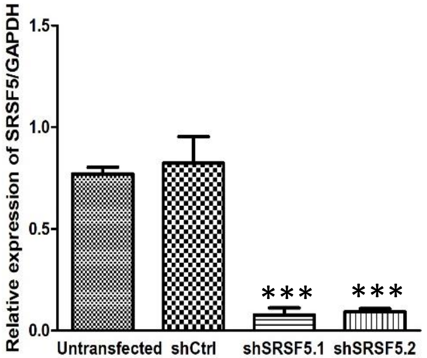

Figure S6

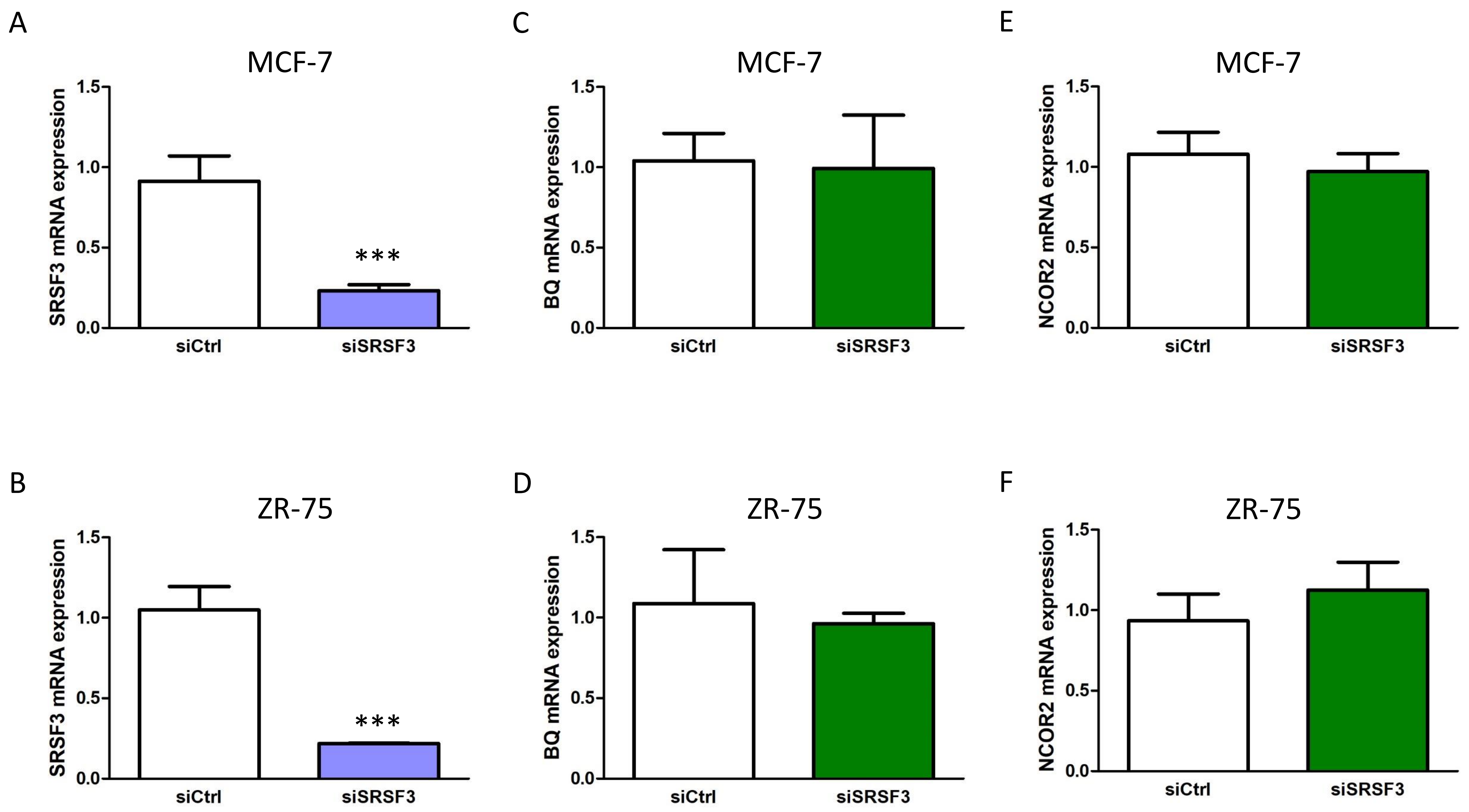

A
MCF-7
SRSF3 mRNA expression
1.5
1.0
0.5
0.0
***
siCtrl
siSRSF3
C
MCF-7
BQ mRNA expression
E
MCF-7
NCOR2 mRNA expression
B
ZR-75
SRSF3 mRNA expression
D
ZR-75
BQ mRNA expression
F
ZR-75
NCOR2 mRNA expression

Figure S7

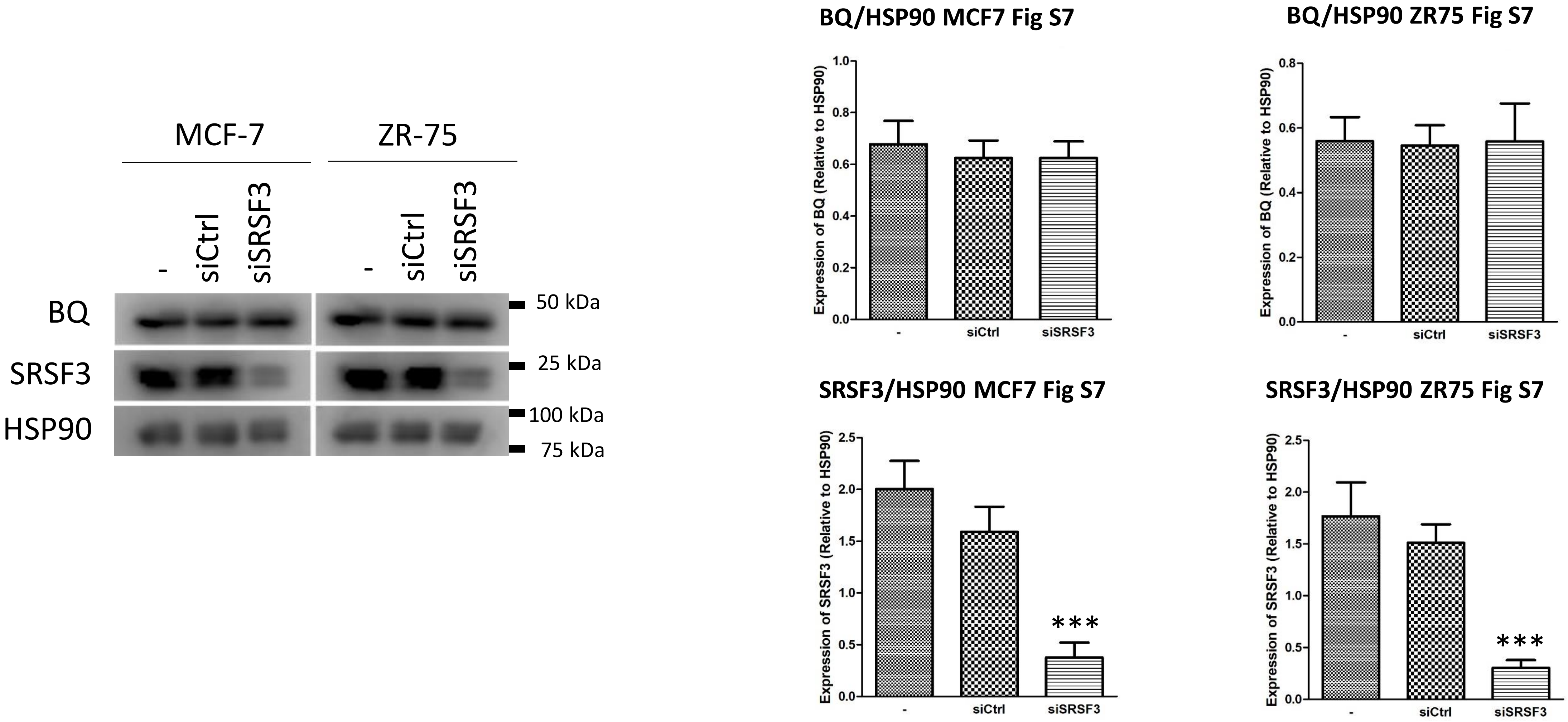
MCF-7
ZR-75
-
siCtrl
siSRSF3
-
siCtrl
siSRSF3
BQ
SRSF3
HSP90
50 kDa
25 kDa
100 kDa
75 kDa
BQ/HSP90 MCF7 Fig S7
Expression of BQ (Relative to HSP90)
1.0
0.8
0.6
0.4
0.2
0.0
-
siCtrl
siSRSF3
BQ/HSP90 ZR75 Fig S7
Expression of BQ (Relative to HSP90)
0.8
0.6
0.4
0.2
0.0
-
siCtrl
siSRSF3
SRSF3/HSP90 MCF7 Fig S7
Expression of SRSF3 (Relative to HSP90)
2.5
2.0
1.5
1.0
0.5
0.0
***
-
siCtrl
siSRSF3
SRSF3/HSP90 ZR75 Fig S7
Expression of SRSF3 (Relative to HSP90)
2.5
2.0
1.5
1.0
0.5
0.0
***
-
siCtrl
siSRSF3

Figure S8

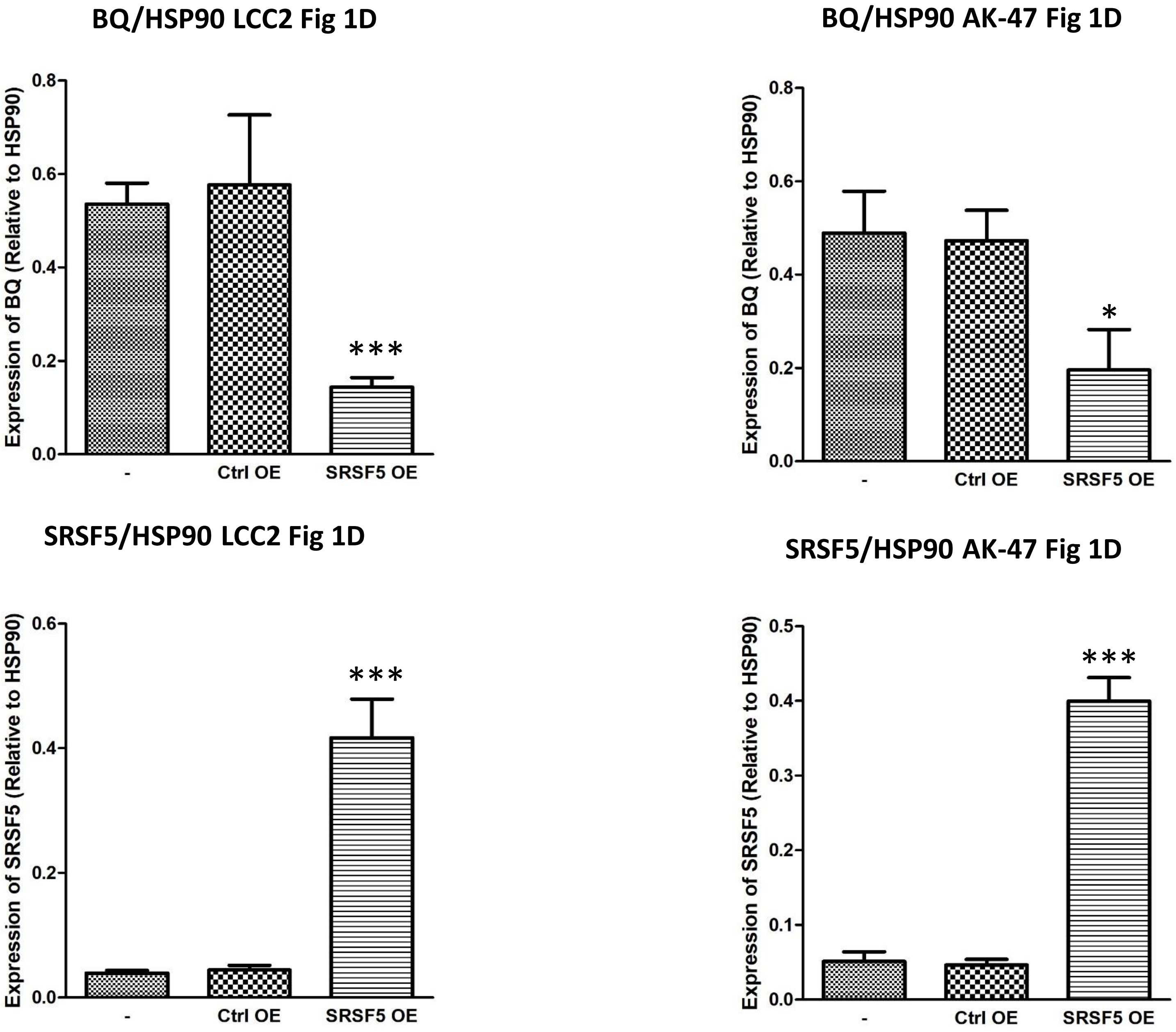
BQ/HSP90 LCC2 Fig 1D
Expression of BQ (Relative to HSP90)
0.0
0.2
0.4
0.6
0.8
***
-
Ctrl OE
SRSF5 OE
BQ/HSP90 AK-47 Fig 1D
Expression of BQ (Relative to HSP90)
0.0
0.2
0.4
0.6
0.8
*
-
Ctrl OE
SRSF5 OE
SRSF5/HSP90 LCC2 Fig 1D
Expression of SRSF5 (Relative to HSP90)
0.0
0.2
0.4
0.6
***
-
Ctrl OE
SRSF5 OE
SRSF5/HSP90 AK-47 Fig 1D
Expression of SRSF5 (Relative to HSP90)
0.0
0.1
0.2
0.3
0.4
0.5
***
-
Ctrl OE
SRSF5 OE

Figure S9

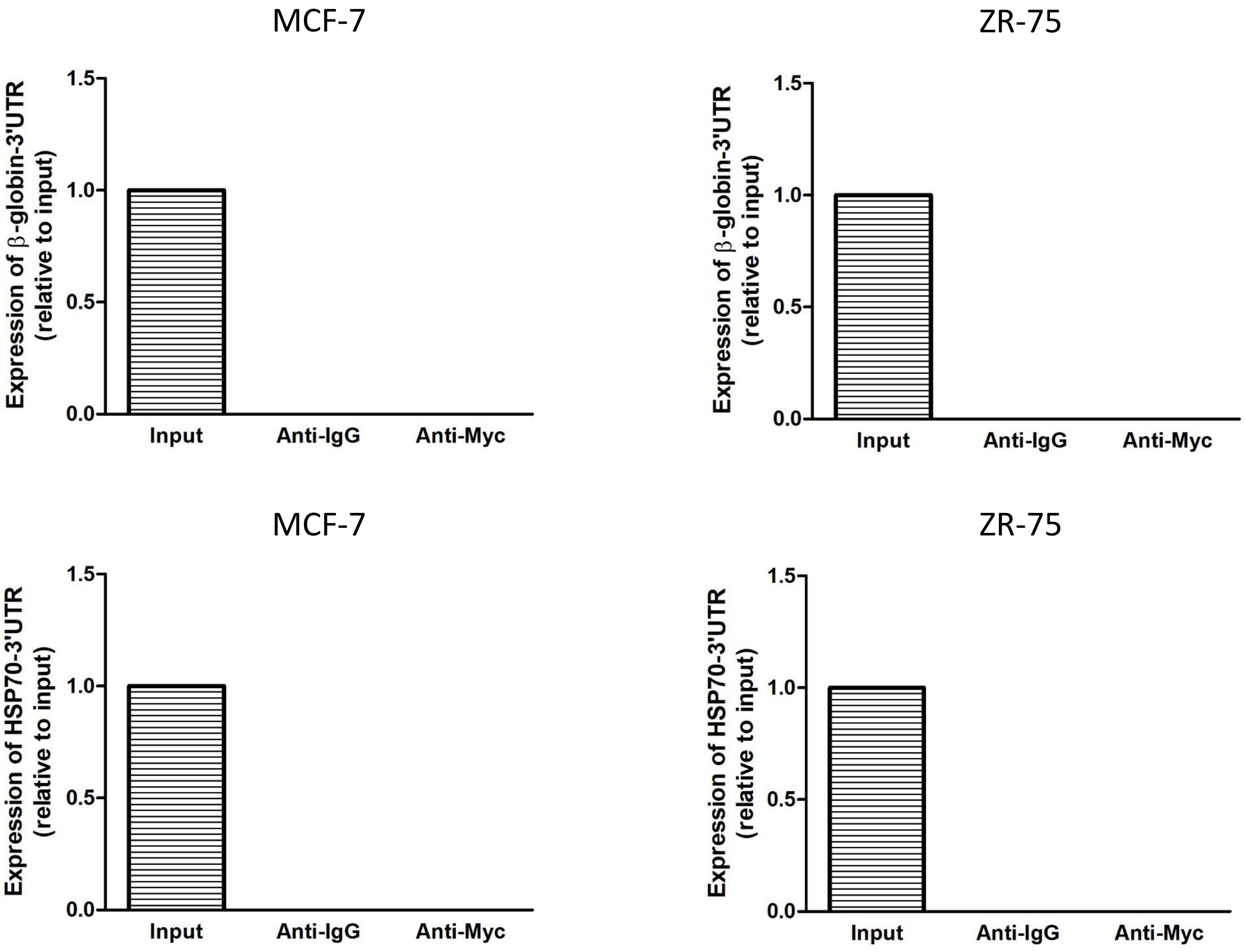

MCF-7
ZR-75
Expression of β-globin-3'UTR (relative to input)
Expression of HSP70-3'UTR (relative to input)
1.5
1.0
0.5
0.0
Input
Anti-IgG
Anti-Myc

Figure S10

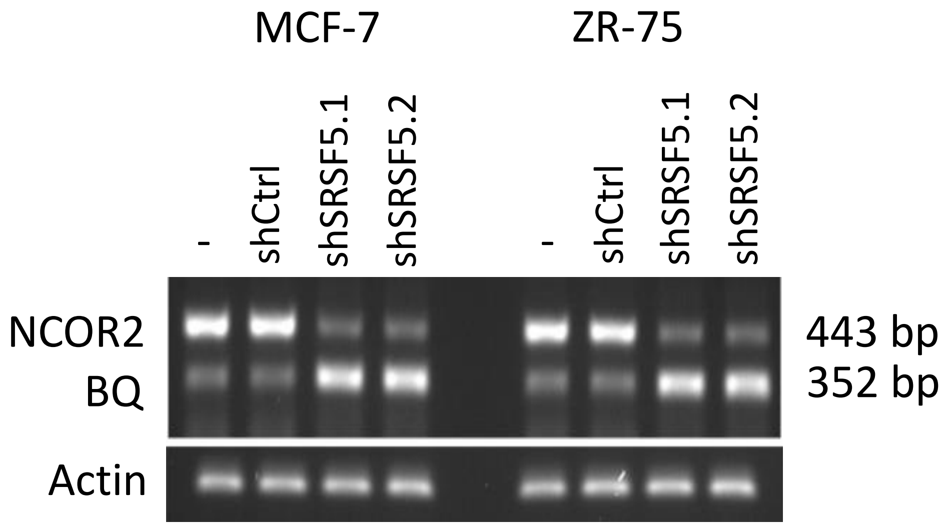
MCF-7
ZR-75
-
shCtrl
shSRSF5.1
shSRSF5.2
-
shCtrl
shSRSF5.1
shSRSF5.2
NCOR2
BQ
443 bp
352 bp
Actin

Figure S11

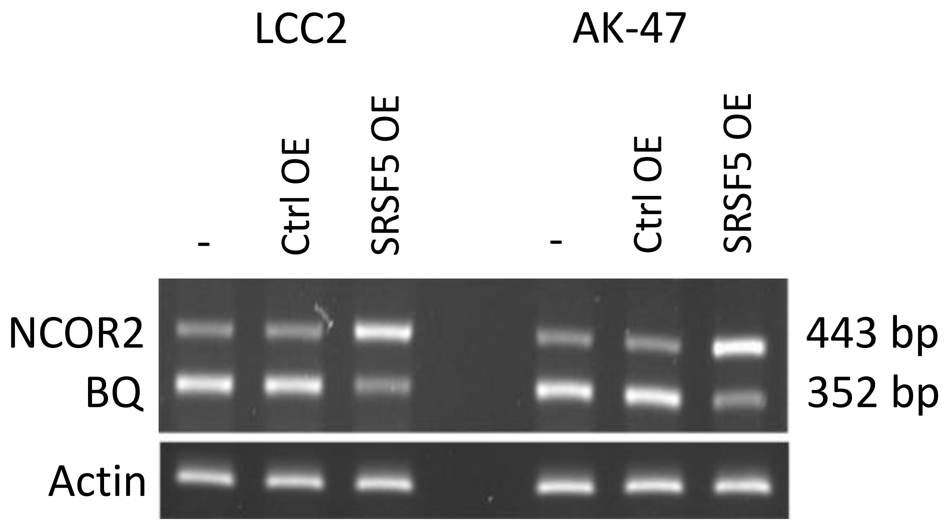

LCC2
AK-47
-
Ctrl OE
SRSF5 OE
-
Ctrl OE
SRSF5 OE
NCOR2
BQ
Actin
443 bp
352 bp

Figure S12

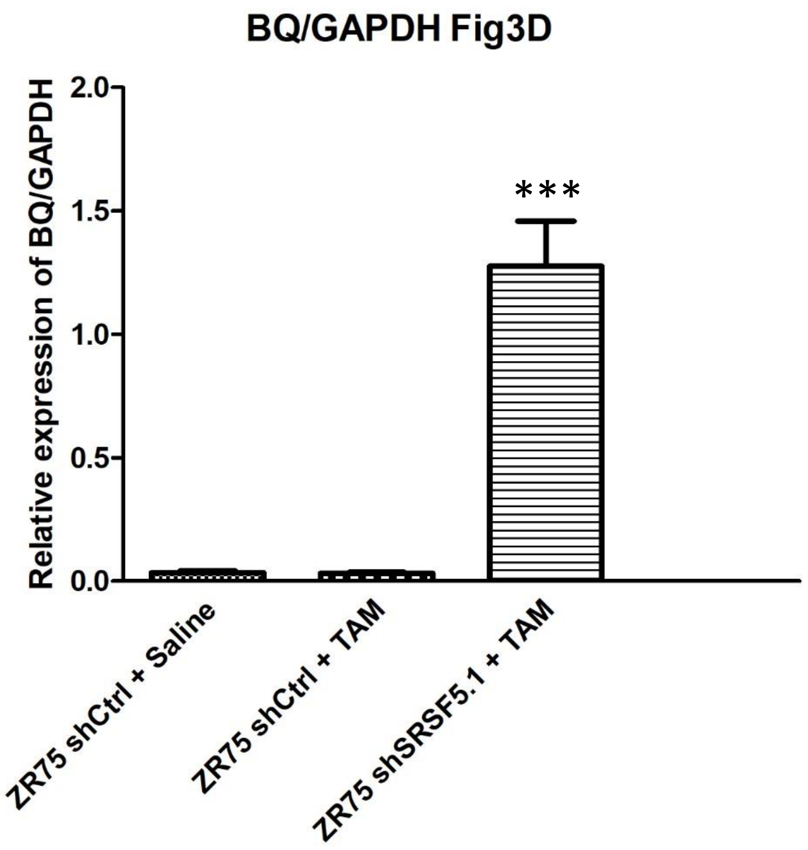

BQ/GAPDH Fig3D
Relative expression of BQ/GAPDH
2.0
1.5
1.0
0.5
0.0
***
ZR75 shCtrl + Saline
ZR75 shCtrl + TAM
ZR75 shSRSF5.1 + TAM

Figure S13

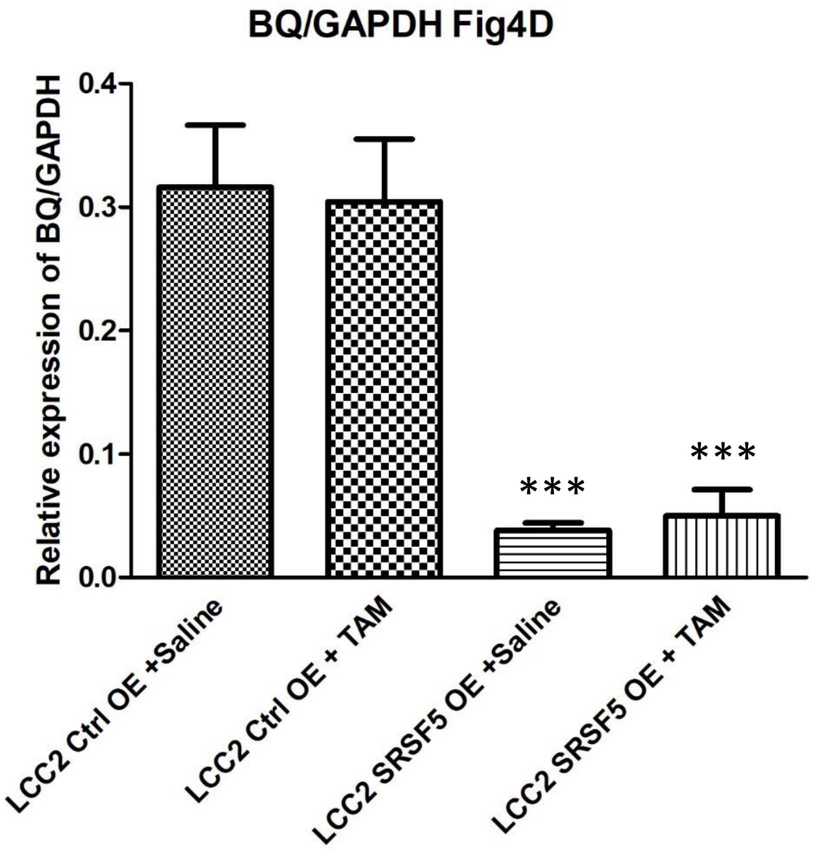

BQ/GAPDH Fig4D
Relative expression of BQ/GAPDH
0.4
0.3
0.2
0.1
0.0
***
***
LCC2 Ctrl OE +Saline
LCC2 Ctrl OE + TAM
LCC2 SRSF5 OE +Saline
LCC2 SRSF5 OE + TAM

Figure S14

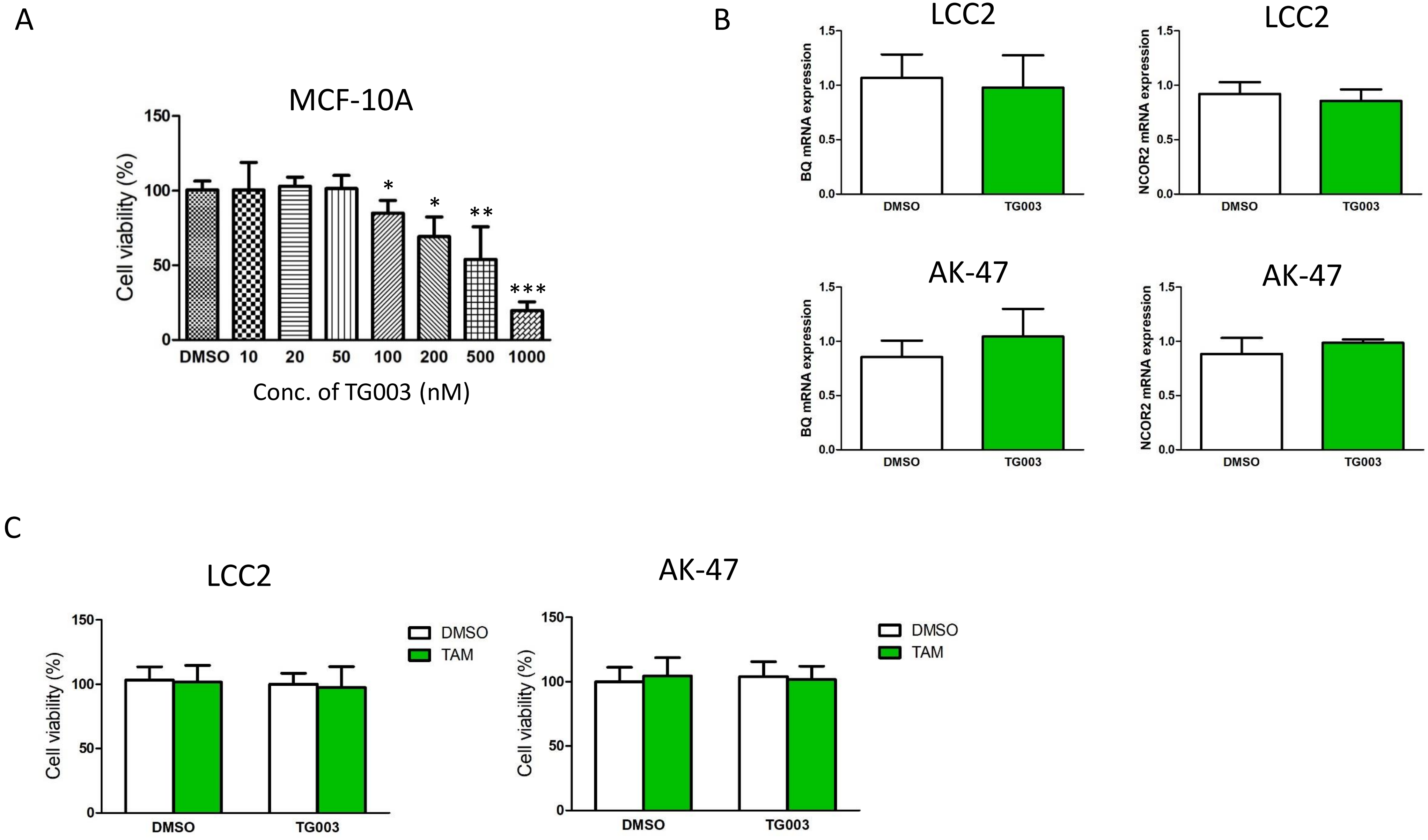
A
MCF-10A
Cell viability (%)
150
100
50
0
*
*
**
***
DMSO
10
20
50
100
200
500
1000
Conc. of TG003 (nM)
B
LCC2
BQ mRNA expression
NCOR2 mRNA expression
1.5
1.0
0.5
0.0
DMSO
TG003
AK-47
C
LCC2
AK-47
Cell viability (%)
DMSO
TAM
DMSO
TG003

Figure S15

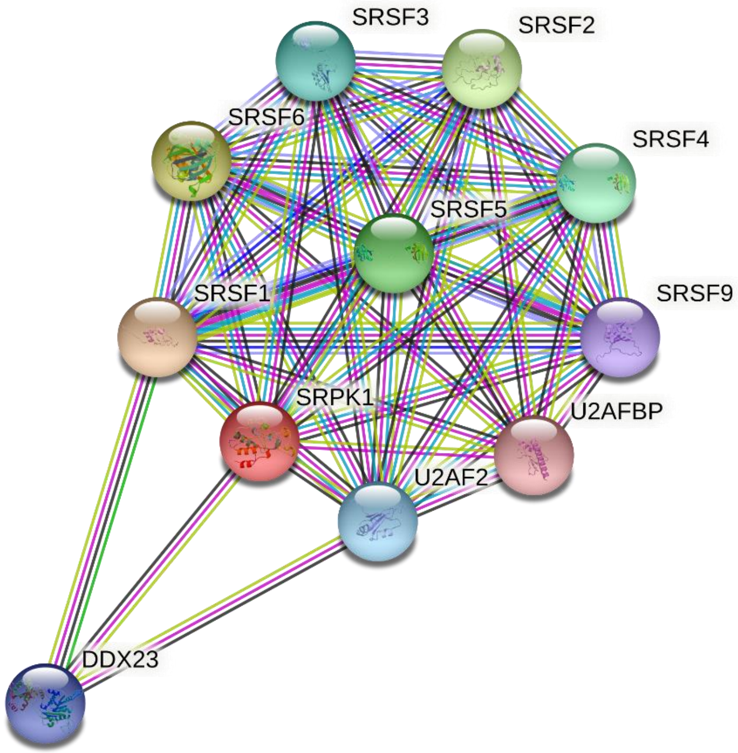

SRSF3
SRSF2
SRSF6
SRSF4
SRSF5
SRSF1
SRSF9
SRPK1
U2AFBP
U2AF2
DDX23

Figure S16

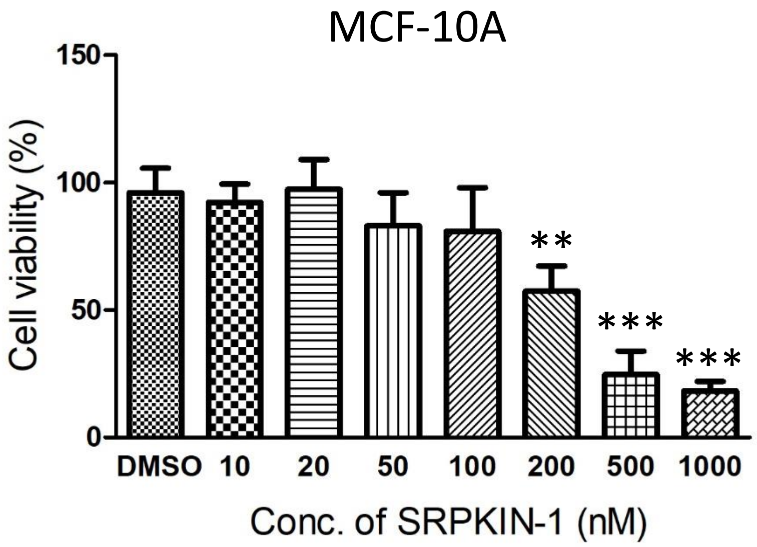
MCF-10A
150
100
50
0
Cell viability (%)
**
***
***
DMSO
10
20
50
100
200
500
1000
Conc. of SRPKIN-1 (nM)

Figure S17

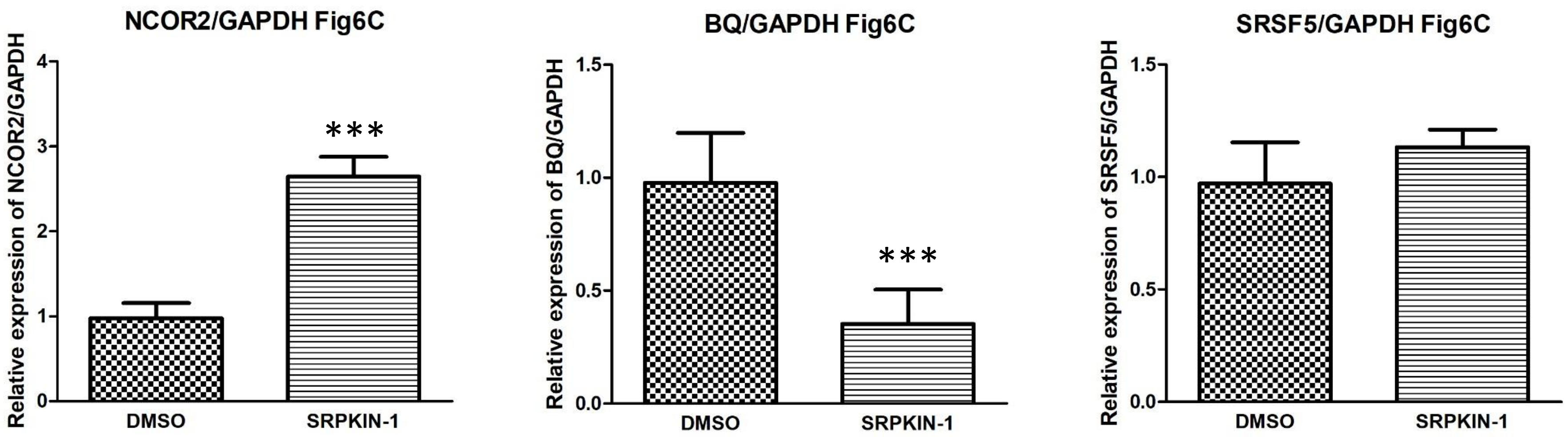
NCOR2/GAPDH Fig6C
Relative expression of NCOR2/GAPDH
0
1
2
3
4
***
DMSO
SRPKIN-1
BQ/GAPDH Fig6C
Relative expression of BQ/GAPDH
0.0
0.5
1.0
1.5
***
DMSO
SRPKIN-1
SRSF5/GAPDH Fig6C
Relative expression of SRSF5/GAPDH
0.0
0.5
1.0
1.5
DMSO
SRPKIN-1

Figure S18

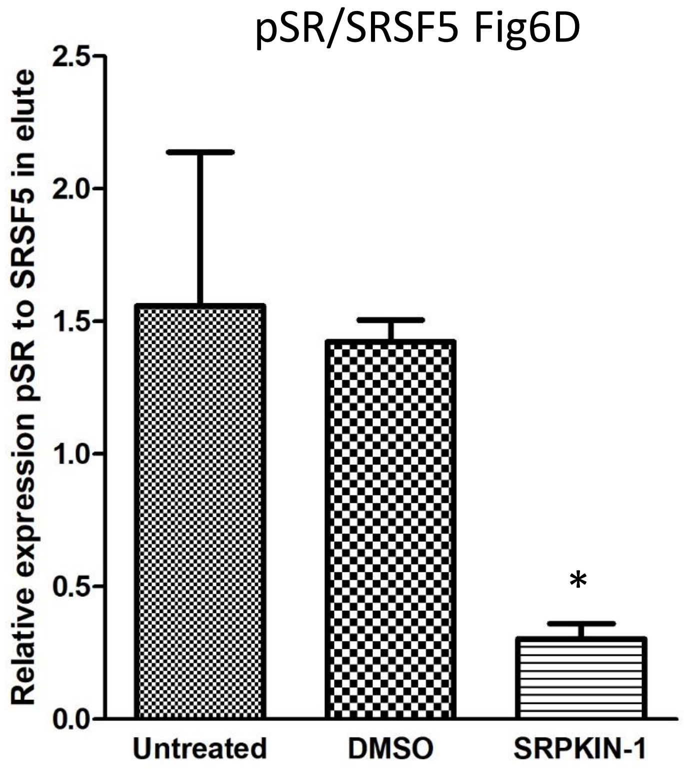

pSR/SRSF5 Fig6D
Relative expression pSR to SRSF5 in elute
2.5
2.0
1.5
1.0
0.5
0.0
*
Untreated
DMSO
SRPKIN-1

Figure 1B uncropped blots

SRSF5 in Figure 1B

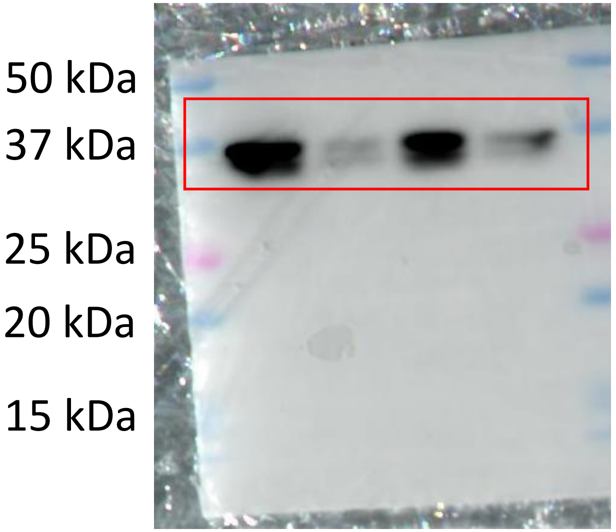

SRSF3 in Figure 1B

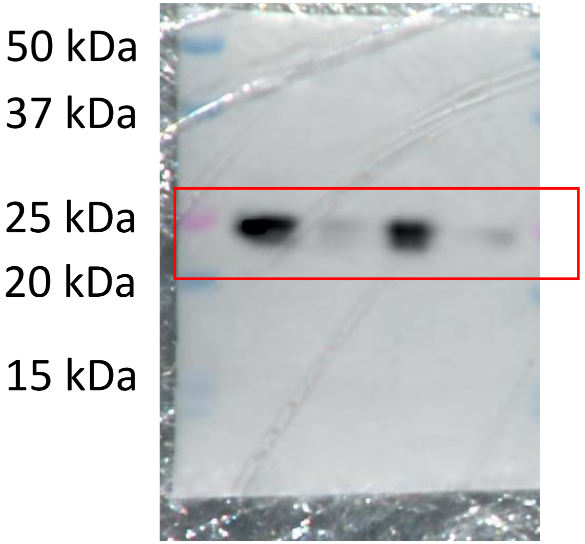

BQ in Figure 1B

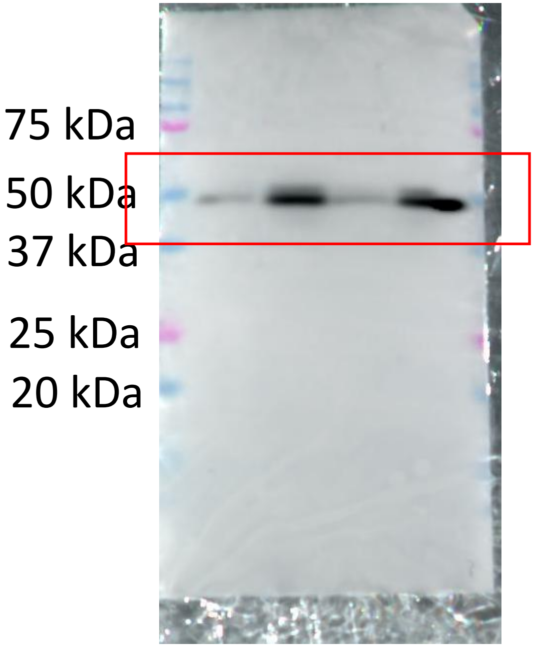

HSP90 in Figure 1B

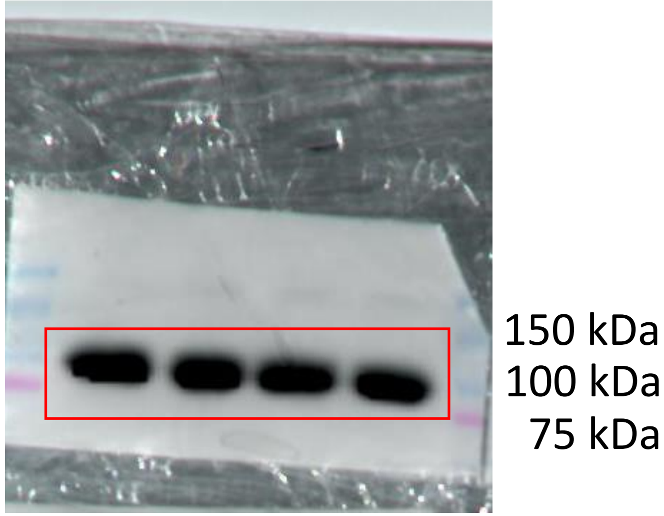

Figure 1C uncropped blots

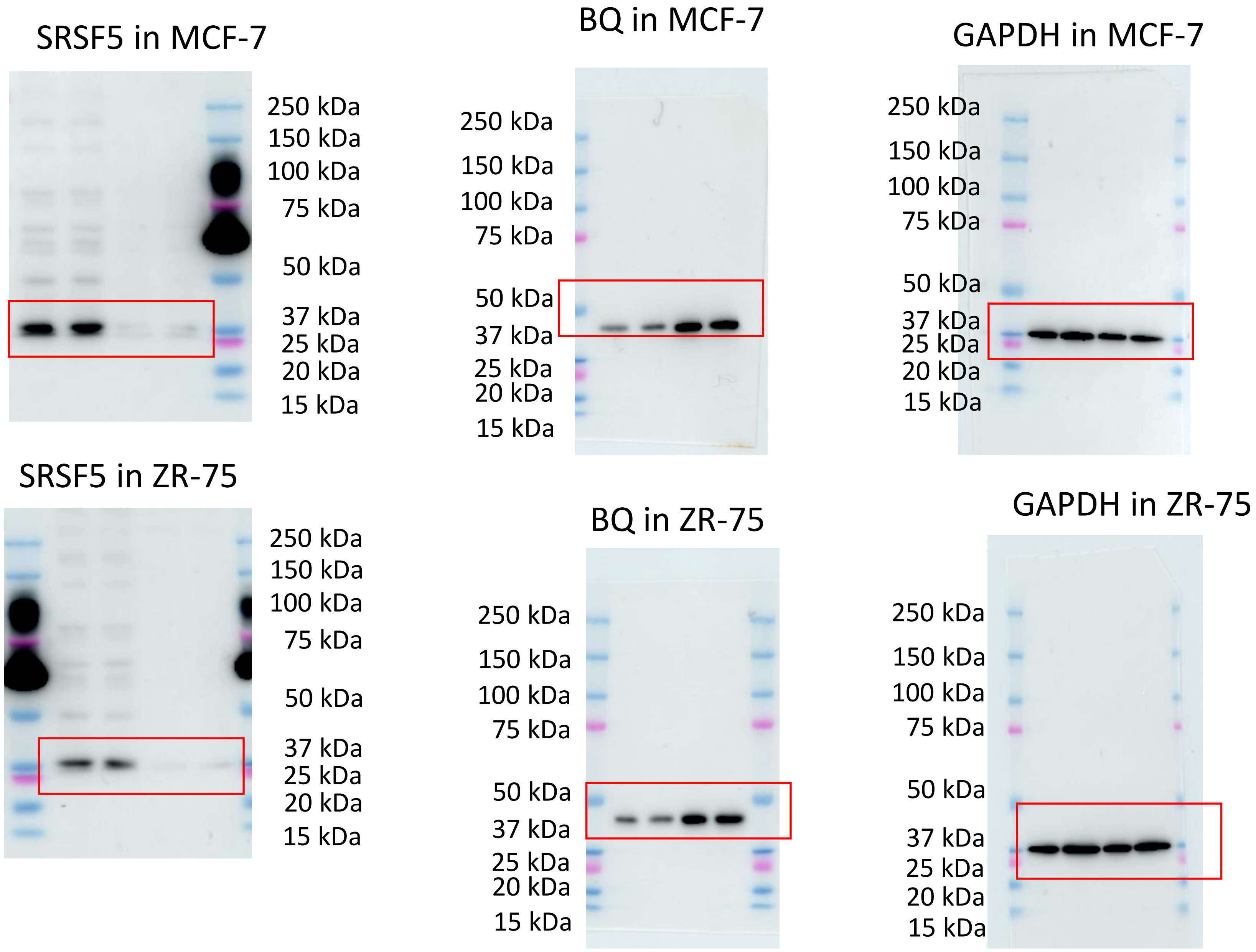

Figure 1D uncropped blots

SRSF5 in LCC2

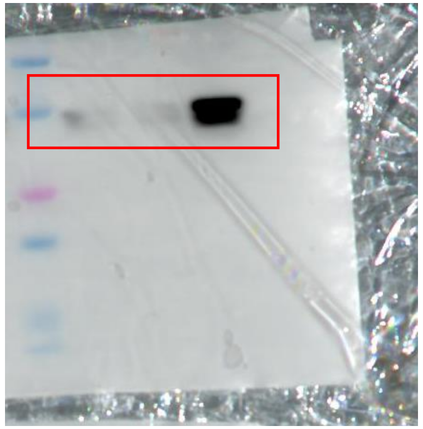

BQ in LCC2

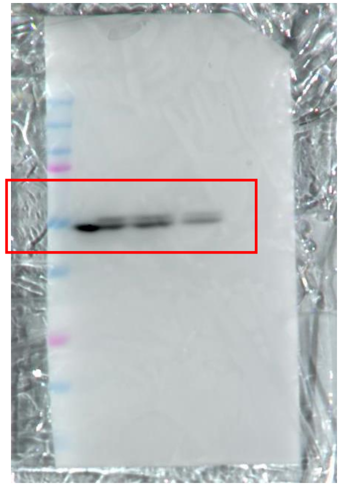

HSP90 in LCC2

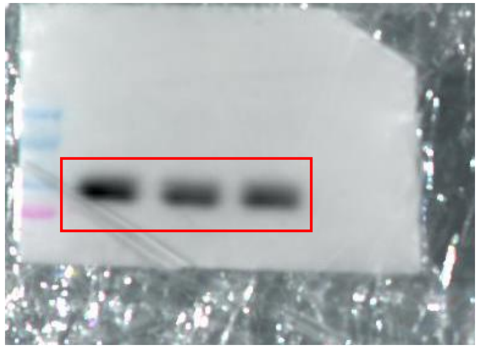

SRSF5 in AK-47

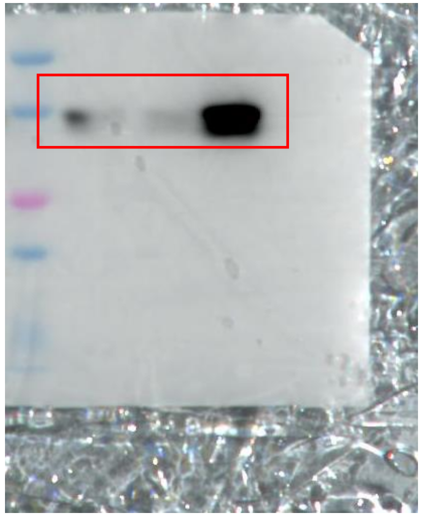

BQ in AK-47

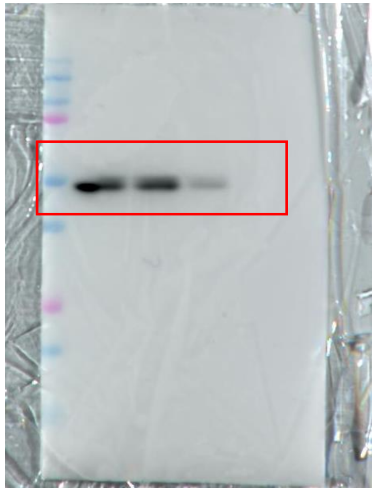

HSP90 in AK47

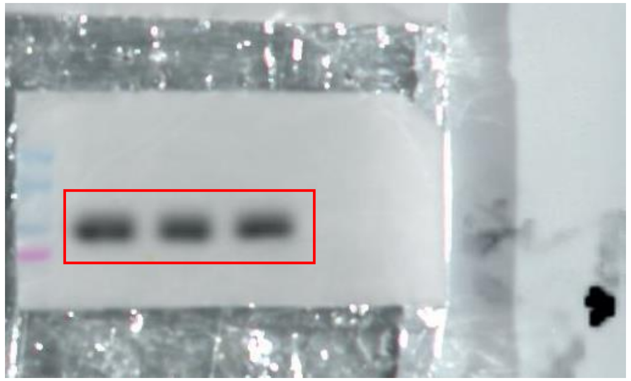

Figure 3D uncropped blots

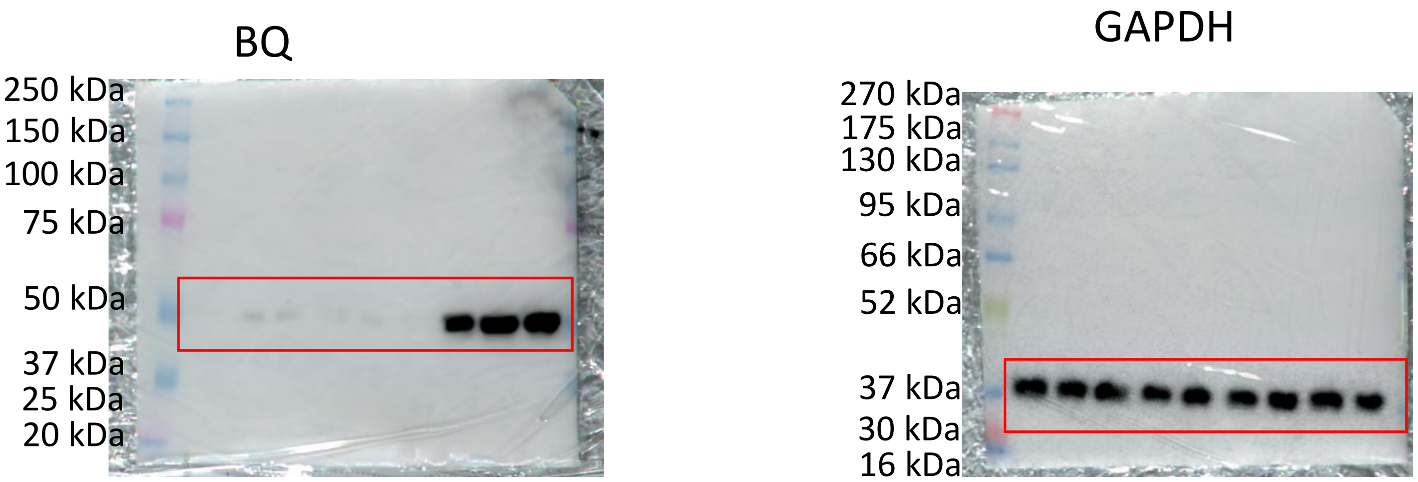

Figure 4D uncropped blots

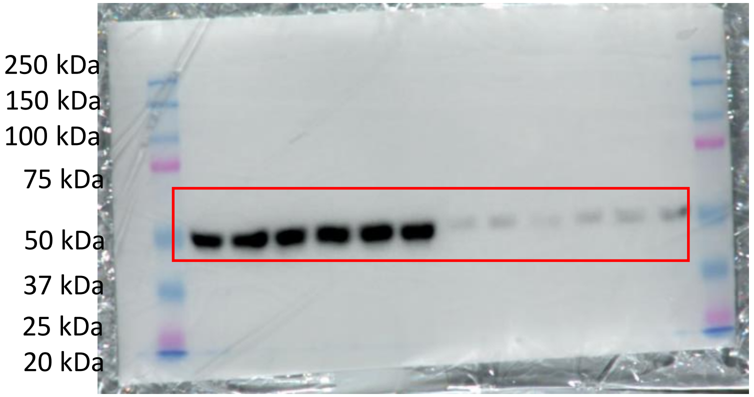

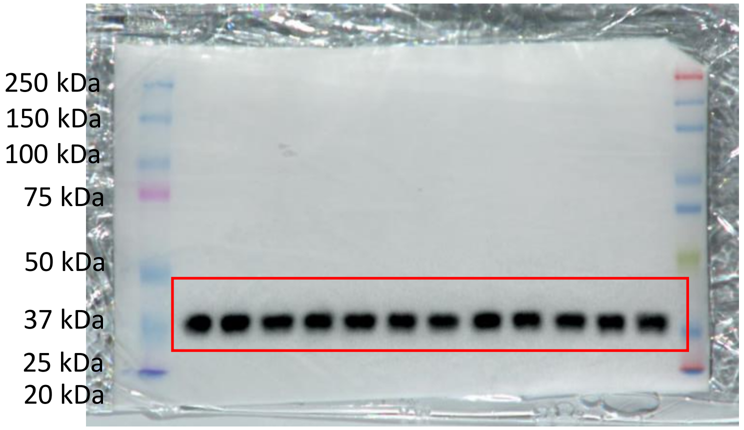

Figure 6A uncropped blots

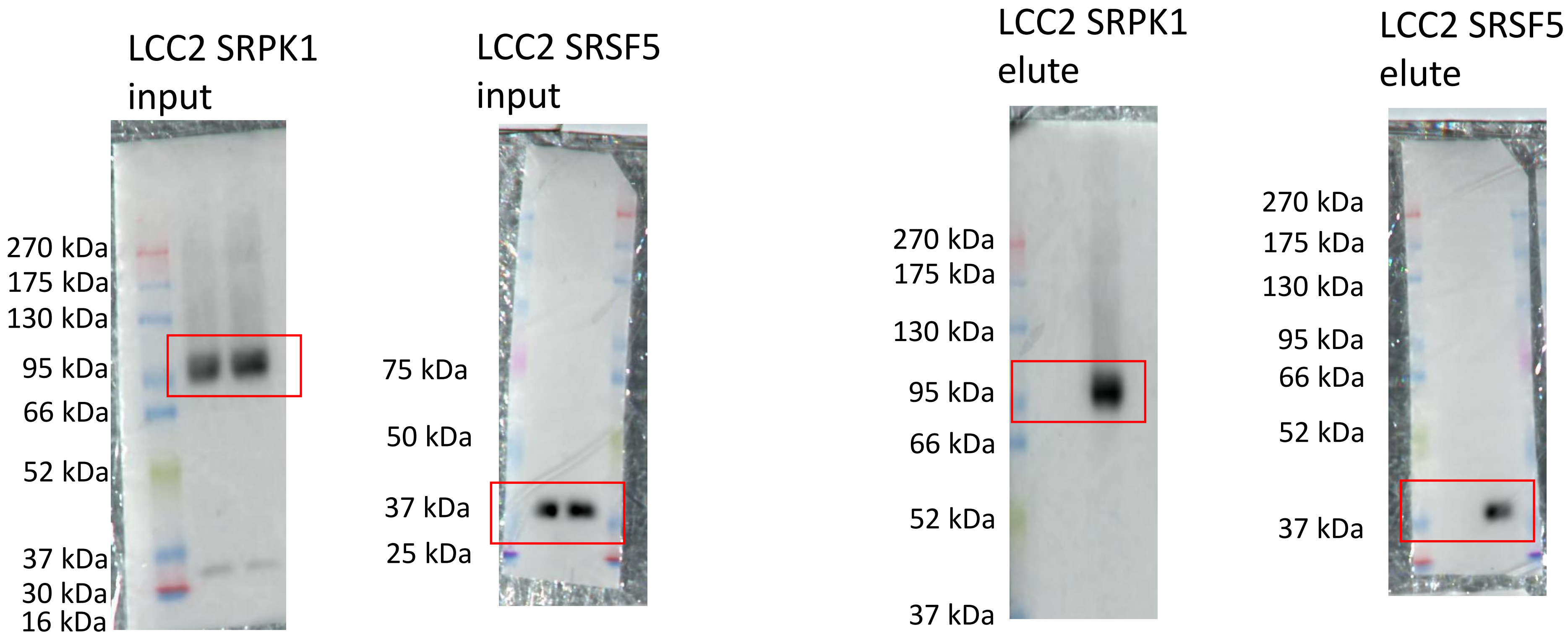

Figure 6C uncropped blots

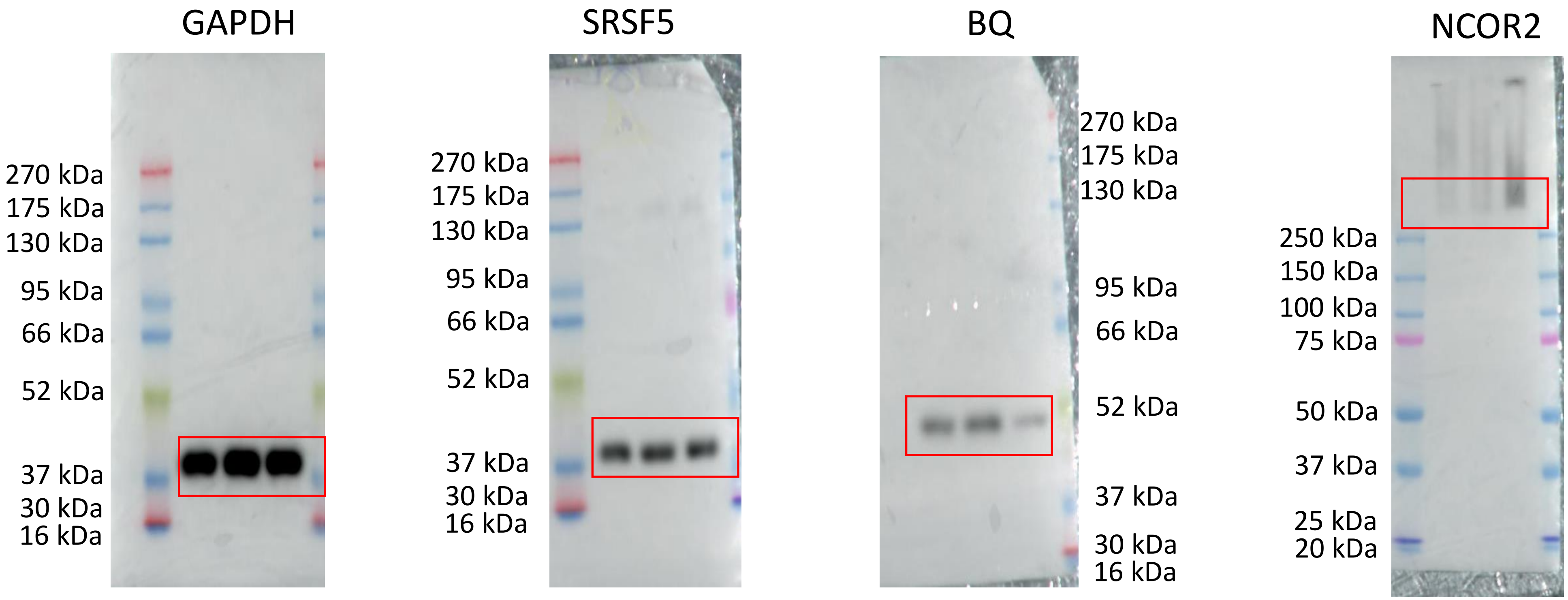

Figure 6D uncropped blots

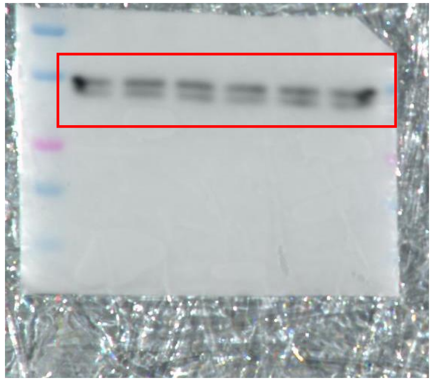

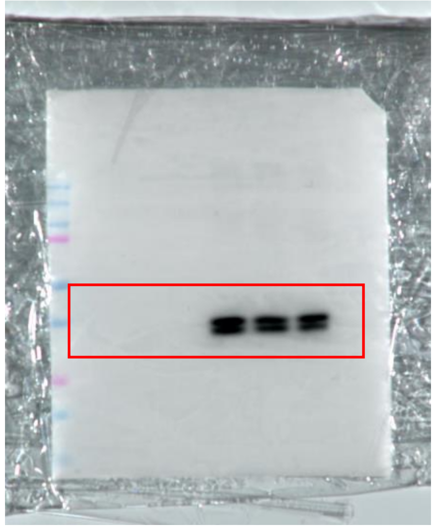

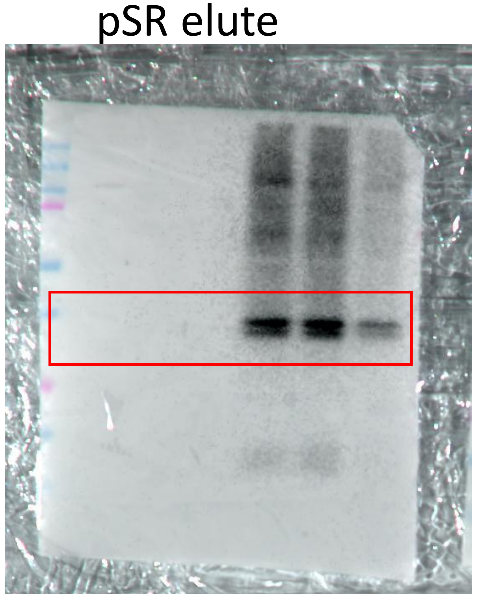

Figure S7 uncropped blots

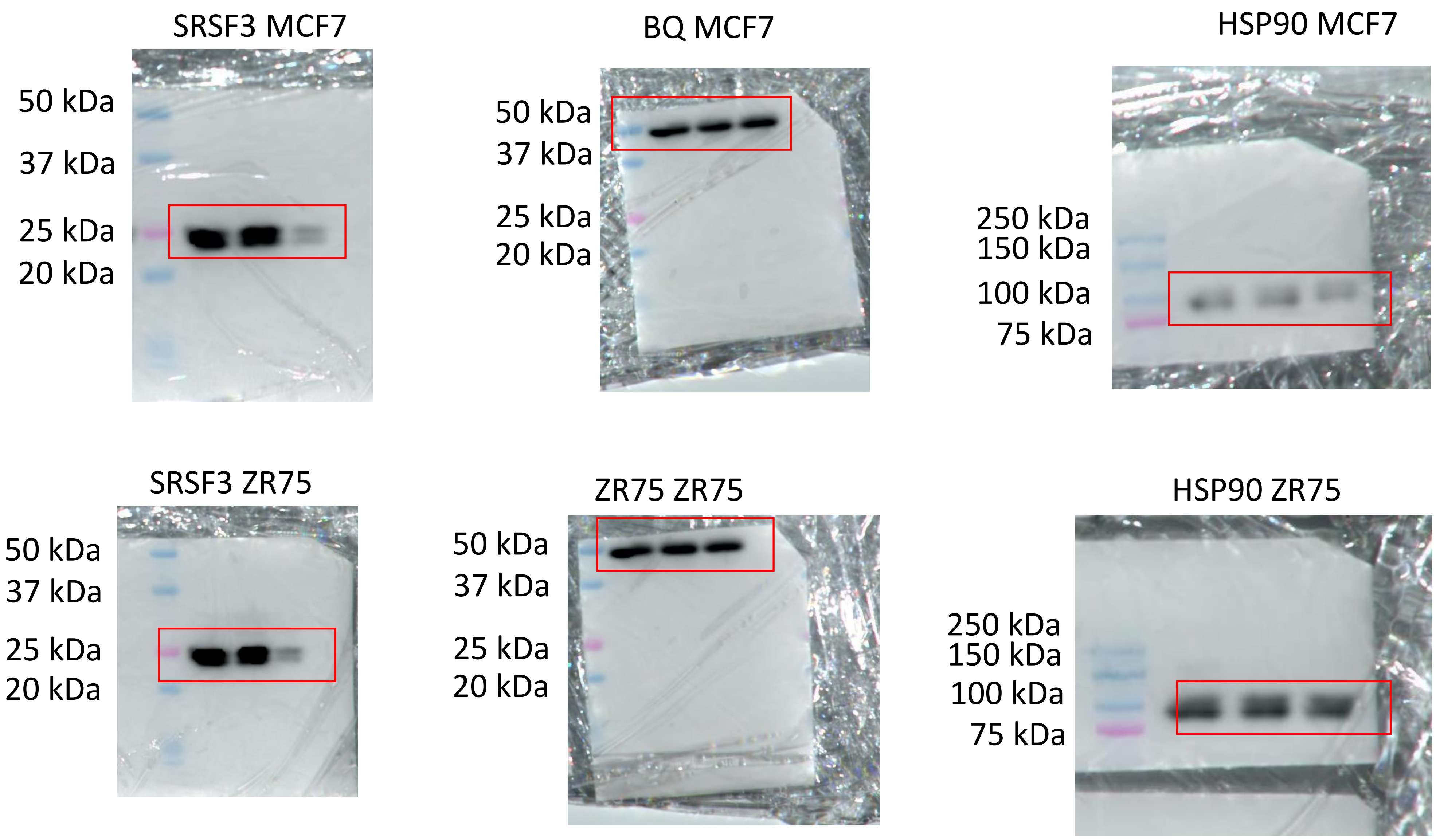

Full agarose gel image for figure S1

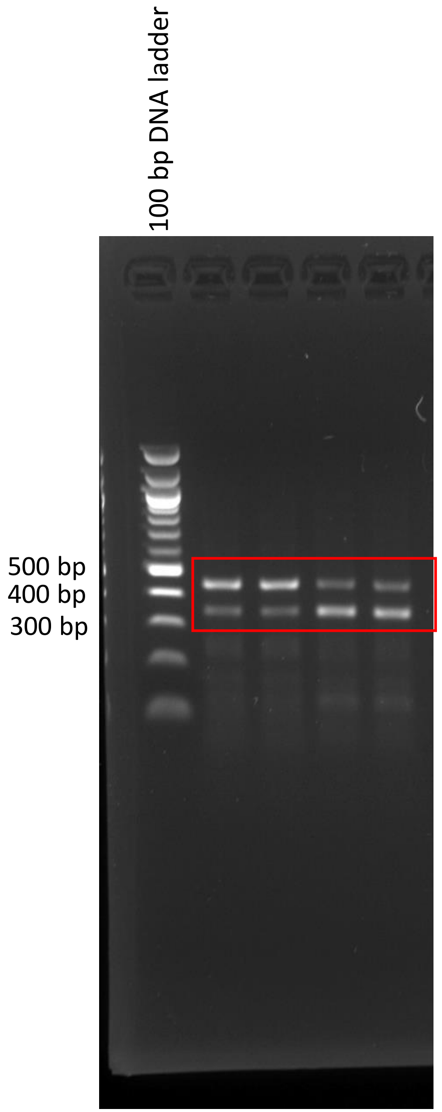

NCOR2 BQ splicing

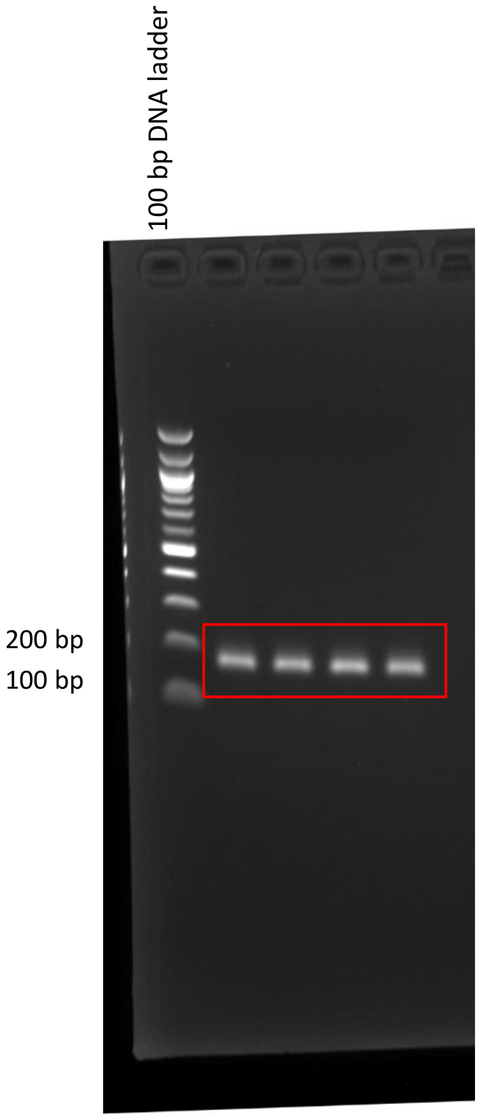

Actin

Full agarose gel image for figure 10

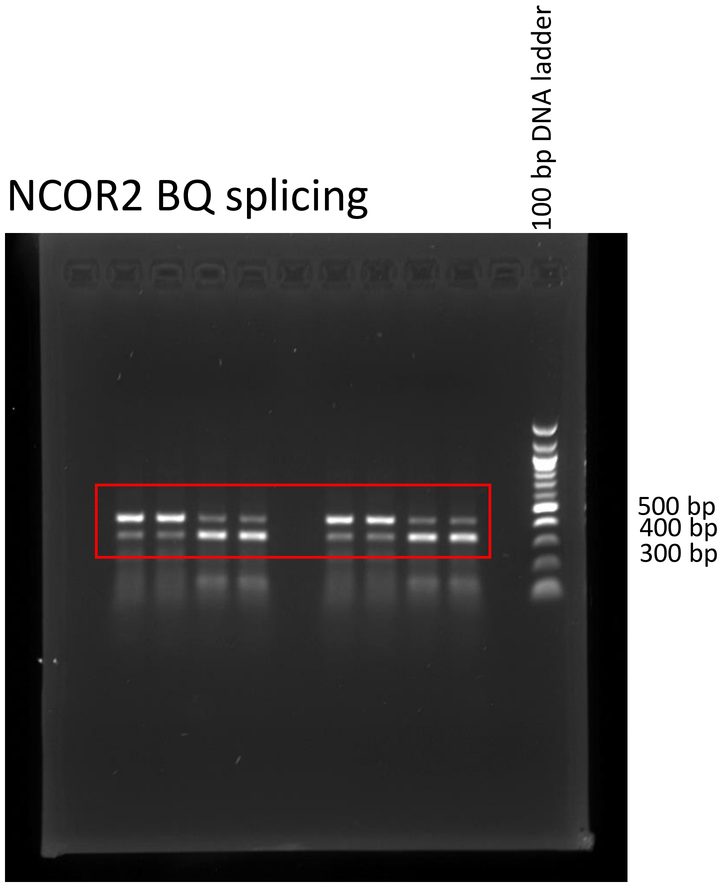

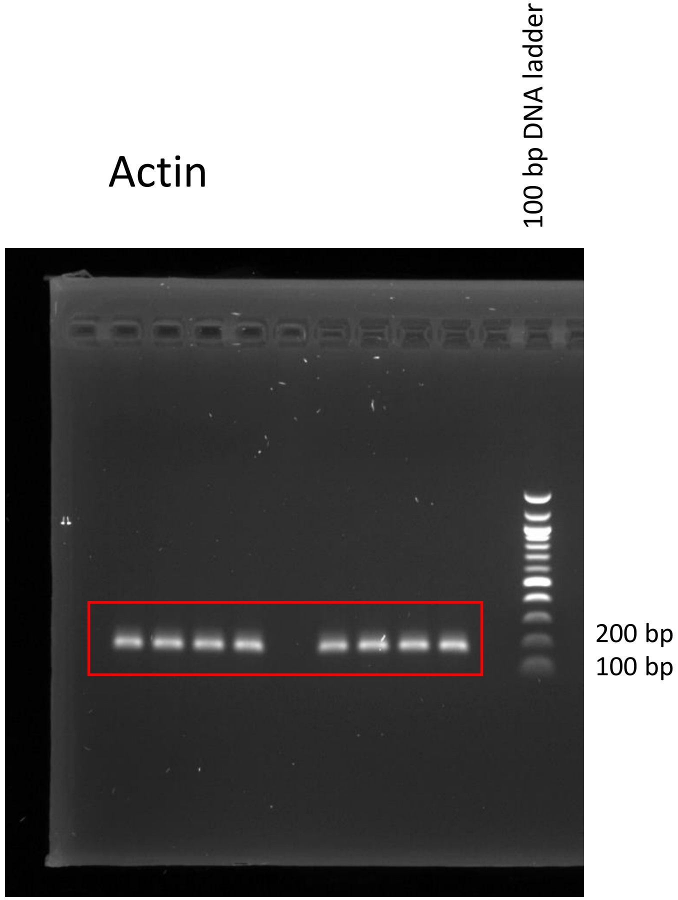

Full agarose gel image for figure S11

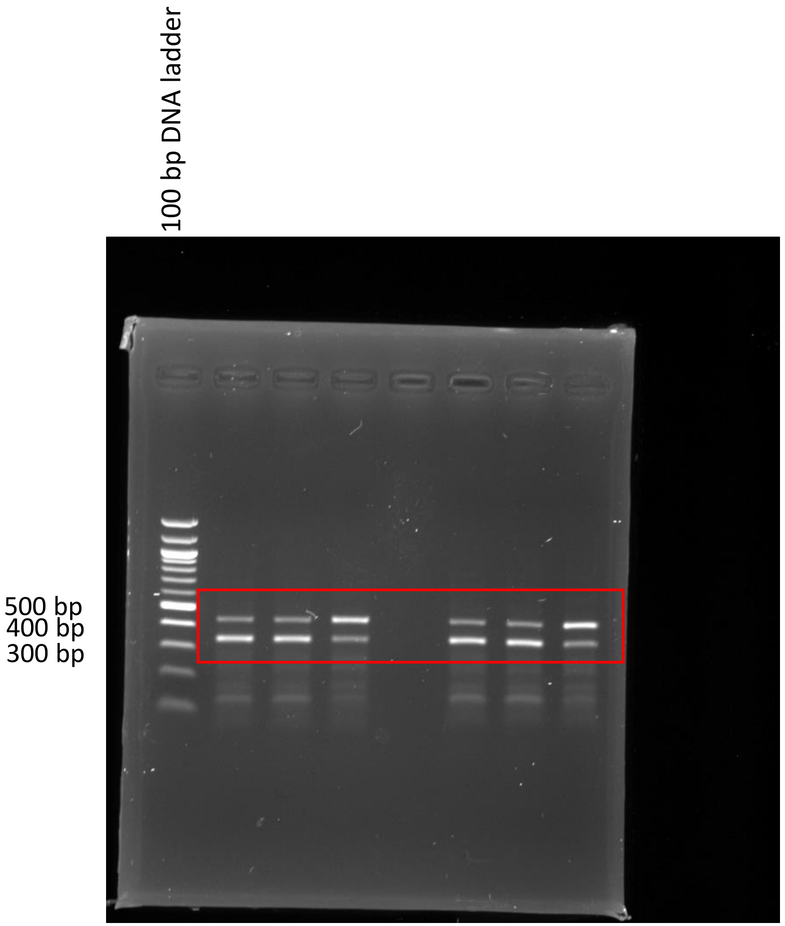

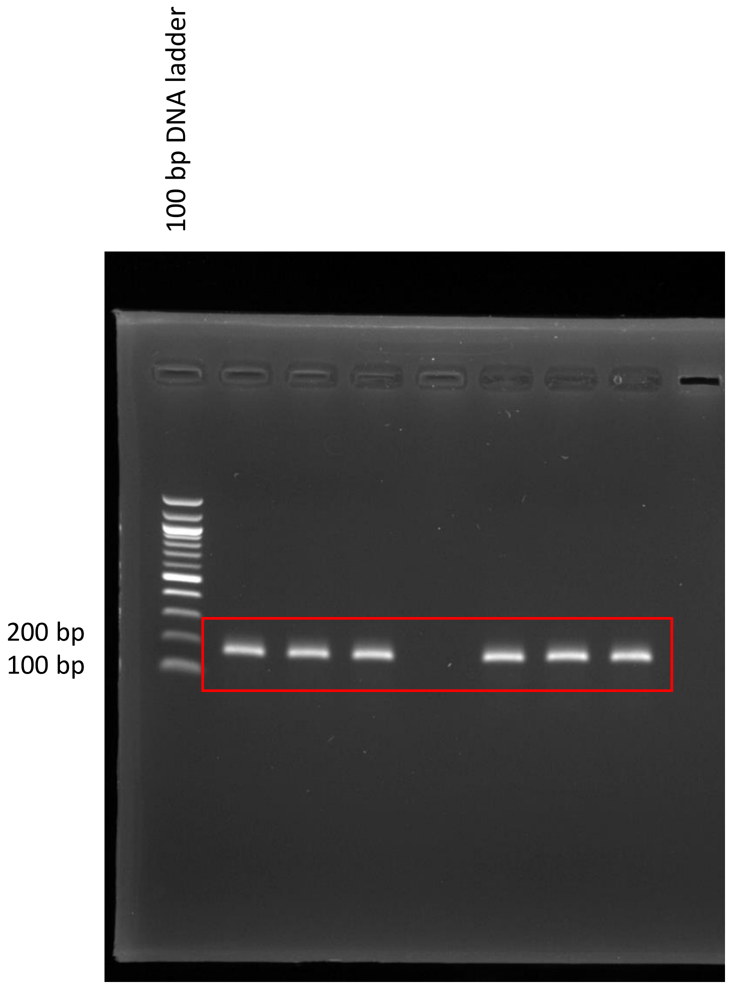

Supplementary figure legends

Figure S1 PCR-based splicing assay of NCOR2 and BQ. The DNA sequence of exons 10-12 of NCOR2 is shown. Using NCOR2-BQ splicing forward and reverse primers would yield two different PCR products with different product sizes, 443 bp and 352 bp for NCOR2 and BQ amplicons, respectively. Actin was the loading control.

Figure S2 Expression levels of SRSF1-12. qPCR was performed. ΔΔCT method was used and MCF-10A was used as the reference. Actin was used as the internal control. Results were shown as mean ± sd from three independent experiments.

Figure S3 Quantification of western blot results in Figure 1B. Results were shown as mean ± SD from four independent experiments. Students' t-test was employed for statistical analysis. *** represents $p < 0.001$.

Figure S4 Knockdown efficiency of shRNA targeting SRSF5. qPCR was employed to determine the expression of SRSF5. Actin was employed as the internal control. ΔΔCT method was used to evaluate the relative gene expression level. Results were shown as mean ± SD from three independent experiments. One-way ANOVA with Bonferroni post hoc test was used to determine the statistical significance between shCtrl and shSRSF5.1 or shSRSF5.2 groups. *** represents $p < 0.001$.

Figure S5 Quantification of western blot results in Figure 1C. Results were shown as mean ± SD from four independent experiments. One-way ANOVA with Bonferroni post hoc test was used to determine the statistical significance between shCtrl and shSRSF5.1 or shSRSF5.2 groups. *** represents $p < 0.001$.

Figure S6 The effect of SRSF3 siRNA. SRSF3 expression in A) MCF-7 and B) ZR-75. BQ expression in C) MCF-7 and D) ZR-75. NCOR2 expression in E) MCF-7 and F) ZR-75. 25 nM of siSRSF3 and siCtrl was employed. qPCR was performed 48 hours post-transfection. Actin was employed as the internal control. ΔΔCT method was used to evaluate the relative gene expression level. Results were shown as mean ± SD from four independent experiments. Students' t-test was employed for statistical analysis. *** represents $p < 0.001$.

Figure S7 SRSF3 knockdown could not affect BQ expression. 25 nM of siSRSF3 and siCtrl was employed. Western blot was performed 48 hours post-transfection. HSP90 was the loading control. The bar charts are the quantification of the western blots. Results were shown as mean ± SD from four independent experiments. Students' t-test was employed for statistical analysis. *** represents $p < 0.001$.

Figure S8 Quantification of western blot results in Figure 1D. Results were shown as mean ± SD from four independent experiments. One-way ANOVA with Bonferroni post hoc test was used to determine the statistical significance between untransfected and SRSF5 OE groups. *, and *** represent $p < 0.05$ and $p < 0.001$, respectively.

Figure S9 SRSF5 could not bind to 3'-UTR of HSP70 and β-globin. MCF-7 and ZR-75 were transfected and overexpressed with myc-tagged SRSF5. Immunoprecipitation was performed using anti-SRSF5 or anti-IgG. RNA associated with SRSF5 was extracted by Trizol reagent. RT-qPCR was performed to detect the expression of 3'-UTR of HSP70 and β-globin. The expression of these candidates in the input fraction was used as reference. 3'-UTR of HSP70 and β-globin were absent in the immunoprecipitants. Results were shown as mean ± SD from three independent experiments.

Figure S10 Knockdown of SRSF5 could enhance BQ expression but reduce NCOR2 expression. PCR-based splicing assay was performed on the control (shCtrl) and SRSF5 knockdown (shSRSF5.1 or shSRSF5.2). Actin was the loading control.

Figure S11 Overexpression of SRSF5 could reduce BQ expression but enhance NCOR2 expression. PCR-based splicing assay was performed on LCC2 and AK-47 stably transfected with pCMV-Myc-SRSF5 (SRSF5 OE) or pCMV (Ctrl OE). Actin was the loading control.

Figure S12 Quantification of western blot results in Figure 3D. Results were shown as mean ± SD from three independent tumours. One-way ANOVA with Bonferroni post hoc test was used to determine the statistical significance compared with the control (ZR-75 shCtrl + Saline).. *** represents $p < 0.001$.

Figure S13 Quantification of western blot results in Figure 4D. Results were shown as mean ± SD from three independent tumours. One-way ANOVA with Bonferroni post hoc test was used to determine the statistical significance compared with the control (LCC2 Ctrl OE + Saline). *** represents $p < 0.001$.

Figure S14 TG003 could not affect BQ expression and TAM resistance. A) the dose-dependent effect of CLK1 inhibitor TG003. MCF-10A was treated with different concentrations of TG003 for 96 hours. Cell viability assay was performed. Results were shown as mean ± SD from four independent experiments. One-way ANOVA with Bonferroni post hoc test was used to determine the statistical significance between DMSO and treatment groups. B) the effect of TG003 on BQ and NCOR mRNA expressions in LCC2 and AK-47. The cells were treated with 50 nM of TG003 for 72 hours. qPCR was performed. Results were shown as mean ± SD from four independent experiments. Students' t-test was employed for statistical analysis. C) TG003 could not reverse TAM resistance. LCC2 and AK-47 were treated with 50 nM of TG003 and 5 µM of TAM for 96 hours. Cell viability assay was performed. Results were shown as mean ± SD from four

independent experiments. Two-way ANOVA with Bonferroni post hoc test was used to determine the statistical significance. *, **, and *** represents $p < 0.05$, $p < 0.01$ and $p < 0.001$, respectively.

Figure S15 Protein-protein interaction network of SRPK1. The information was obtained from STRING database.

Figure S16 The dose-dependent effect of SRPK1 inhibitor SRPKIN-1. MCF-10A was treated with different concentrations of SRPKIN-1 for 96 hours. Cell viability assay was performed. Results were shown as mean ± SD from four independent experiments. One-way ANOVA with Bonferroni post hoc test was used to determine the statistical significance between DMSO and treatment groups. ** and *** represents $p < 0.01$ and $p < 0.001$, respectively.

Figure S17 Quantification of western blot results in Figure 6C. Results were shown as mean ± SD from three independent experiments. Students' t-test was employed for statistical analysis. *** represents $p < 0.001$.

Figure S18 Quantification of western blot results in Figure 6D. SRSF5-Myc and pSR from elute fraction were normalised using SRSF5-Myc from input fraction. The relative expression of normalised SRSF5-Myc and pSR was determined. Results were shown as mean ± SD from three independent experiments. One-way ANOVA with Bonferroni post hoc test was used to determine the statistical significance between untreated and SRPKIN-1 groups. * represents $p < 0.05$.
